# Natural variation of ncHLAII molecules: challenges and perspectives

**DOI:** 10.1038/s41423-022-00910-0

**Published:** 2022-08-26

**Authors:** Miguel Álvaro-Benito

**Affiliations:** Protein Biochemistry, Institute for Biochemistry, Freie Universität Berlin, Berlin, Germany

**Keywords:** Cellular immunity, Biomarkers

Identifying causal genetic variants for a given phenotype improves diagnosis and provides insights for designing therapies. However, defining the link and/or contribution to a phenotype of rare variants or those with an indirect contribution to disease remains challenging. These variants, however, are key for personalized and precision medicine strategies [1]. In this context, human leukocyte antigens of class II alleles (HLAIIs) facilitate activation of CD4^+^ T cells and are directly linked to various phenotypes. Non-classical HLAII (ncHLAII) molecules modulate HLAII function and their impact on T-cell responses. The contribution of ncHLAII to phenotypes has been investigated in knockout *vs*. wild-type cellular and animal models, and recent studies have shown that genetic variants altering the protein-coding regions of these molecules contribute to tuning of HLAII function [2]. These findings suggest that ncHLAII natural variants may play a key role in modulating immune mechanisms that affect human phenotypes.

**Fig. 1:**
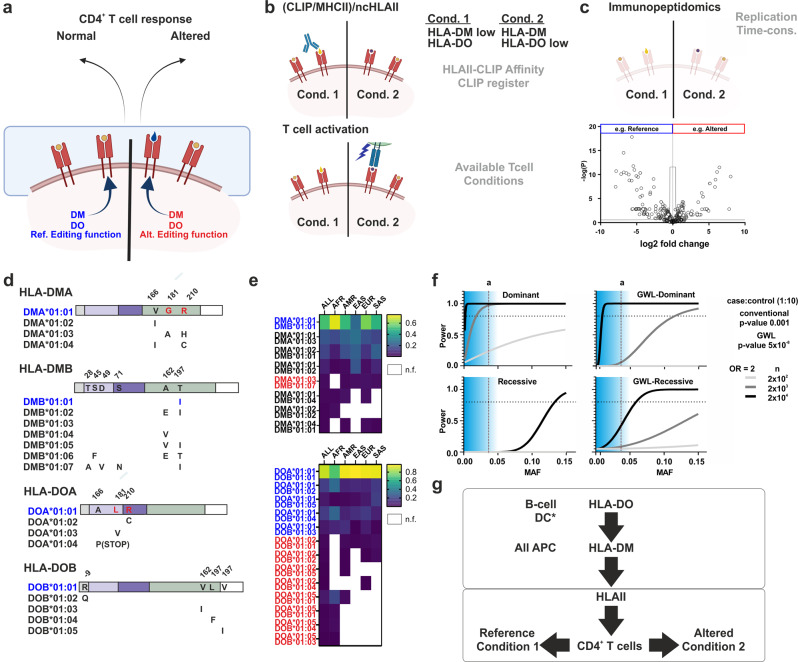
**a** ncHLAII genetic variation modulates the display of distinct peptide repertoires and thus directly influences CD4^+^ T-cell responses. **b** Cellular models used to determine ncHLAII function based on the detection of either reporter or antigenic peptide presentation by flow cytometry (up) or T-cell activation assays (low). The main disadvantages (gray) of each type of experiment are indicated on the right side. The known expected outcomes for altered DM or DO function in the case of the detection of (CLIP/HLAII)/ncHLAII ratios are also depicted. **c** Immunopeptidome analysis emerges as a new methodology that allows for a broad overview of the impact of ncHLAII function in which the whole profile of peptides is determined. As in (**b**), the main disadvantages (gray) are indicated on the right side. **d** Currently described ncHLAII alleles in IPD/IMGT-HLA. **e** Haplotypes described for ncHLAII molecules and their frequency across the different populations of 1000 Genome Project. Reference allotypes and those with similar functions as the reference, as described by experimental assays, are shown in blue. The allotypes indicated in red have experimentally proven altered function. n.f. not found. **f** Statistical power achieved for different models of contribution of a given variant according to the minor allele frequency (MAF) tested. The horizontal dashed line represents the 0.8 (power) threshold considered for reporting valid associations. The vertical dashed line represents the MAF of the DM allotype (**a**) with altered function for illustration. The shaded area represents the range of natural ncHLAII variants. Dominant and recessive models under candidate gene analysis or genome wide-like (GWL) conditions are shown. An odds ratio of 2 is exemplarily chosen as to be in the range of HLAII impact on some autoimmune conditions (Legend: Power calculations under different conditions were retrieved in R using the “genpwr“ package). **g** Altered peptide repertoires are regarded as the contribution of ncHLAII variations to any phenotype. DO is only known to alter HLAII peptide repertoires by blocking DM function in certain cell types and stages. DM directly alters these repertoires in all APCs. The consequence of altered ncHLAII functions is altered peptide repertoires that yield specific T-cell responses predisposing toward or protecting against a given phenotype.

HLA-DM (DM) acts as a chaperone that facilitates peptide exchange from classical HLAII molecules selecting long-life peptide-HLAII complexes in all professional antigen-presenting cells. HLA-DO (DO), on the other hand, binds tightly to DM, chaperoning its function as a competitive inhibitor in a cell and developmental stage-dependent manner [2]. Thus, not only DM but also DM-DO interplay defines the pool of peptides displayed for immune surveillance (Immunopeptidome). The immunopeptidome displayed for CD4^+^ T-cell surveillance influences T-cell development and responses to pathogens and also impacts mechanisms directly related to disease and immune therapy outcomes (Fig. 1a). Interestingly, murine models of altered function of the corresponding ncHLAII homologues exhibit autoimmune [2, 3] and viral-resistant phenotypes [4]. Despite these findings, addressing any potential impact of ncHLAII function and/or variants of these molecules to disease in humans remains challenging. Both, the difficulties in defining reference and altered DM and/or DO function, as well as the lack of genetic knowledge, represent the most important obstacles to accomplish this goal.

Defining altered function for ncHLAII molecules poses a challenge on its own. Indeed, the large diversity of HLAII molecules and their different behavior towards DM function makes it extremely difficult to generalize any statement. There are cellular models focusing on the display of reporter peptide(s) depending on ncHLAII function, which are detected either by flow cytometry or by measuring activation of a T-cell clone or hybridoma (Fig. 1b). Assessment of (CLIP/MHCII)/ncHLAII ratios by flow cytometry is the most widely used assay, as CLIP is the placeholder peptide that chaperones the groove of virtually all MHCII molecules. Nevertheless, certain HLAIIs poorly bind CLIP or may not bind the epitope detected by the CerCLIP antibody (standard in these assays). Unfortunately, there is no such type of assay whereby a single reporter T-cell tool could be used on different HLAIIs. Importantly, these experimental models are mainly based on ncHLAII overexpression and should be carefully interpreted. Recombinant molecules are also used in vitro together with reporter peptides. In this case, binding or dissociation experiments (presence *vs*. absence of ncHLAII), in conjunction with protein‒protein interaction determinations [5], can yield relevant molecular insights. Together, these methods are essential for prioritizing functional studies but their restrictive nature (e.g., one candidate epitope) may bias the interpretation of altered ncHLAII function. The application of immunopeptidomics has contributed to overcoming the narrow view of ncHLAII function and accessing the global picture of the peptide repertoires displayed under different conditions. Moreover, this methodology provides additional cellular insights. To overcome the low replication nature of LC-ESI, typically used in immunopeptidomics, we implemented a label-free quantification strategy that enables reliable comparison between different conditions [6, 7] (Fig. 1c). Next steps towards improved experimental models should seek simulating HLAII complexity and ncHLAII expression levels.

The lack of information at the genetic level represents a second important challenge for determining the potential impact of ncHLAII variation on health and disease. To date, the IPD-IMGT/HLA [8] compiles a handful of variants for DM and DO (Fig. 1d). We hypothesized and validated that a certain DMA allele has functional consequences [5] and later proved that this allele is in strong linkage with one particular DMB allele [7], we further extended this type of analysis to DO together with Graves and collaborators [9]. These efforts revealed that based on available IPD-IMGT/HLA information, there are at least 8 haplotypes for DM and 13 for DO, with specific distributions across the different populations of the 2504 individuals of 1000 Genomes Project (Fig. 1e). However, there has been no dedicated update on DM and DO genetic variation since the early 2000s [2]. Therefore, the question of whether we are ignoring potentially relevant ncHLAII variants remains. Future studies may benefit from considering the impact of other types of variants that affect gene expression, transcription, and/or splicing.

From a genetic perspective, Genome Wide, as well as conventional candidate-gene Association Studies, have the potential to nail down low-frequency variant contributions to disease if certain conditions are met (e.g. Fig. 1f, DM haplotype with altered function “a”). These studies would provide relevant insights for the contribution of ncHLAII variation to disease or phenotypes in terms of odds ratio (for a given variant to be present in a phenotype), model (recessive to dominant) or dependency on HLAII. However, our current view on natural variation of ncHLAII to a phenotype is limited to one DO-homologue variant in mice showing a recessive and independent association with a viral-resistant phenotype [4]. Overcoming this lack of knowledge requires dedicated genetic association studies considering the biological function of ncHLAII molecules (Fig. 1g).

Recently, Meurer and collaborators have shown that DM function imposes immunopeptidome divergences linked to CD4^+^ T-cell allo-responses in experimental settings related to hematopoietic stem cell transplantation (HSCT) [10]. Together with, and in perspective of the proven impact of natural DM variants for altered immunopeptidomes [7] it is conceivable hypothesizing  the impact of ncHLAII variants on allo-responses in HSCT besides other phenotypes. In conclusion, the increasing evidence, and the interest in understanding disease-relevant mechanisms in a personalized manner justifies further efforts to score the impact of ncHLAII variants on health and disease. Importantly, solving the challenges described here calls upon coordinated efforts for improving experimental models and genetic association studies.
